# Accessibility of pertussis vaccine immunization services in Hubei Province from a supply–demand coupling perspective

**DOI:** 10.3389/fpubh.2025.1669984

**Published:** 2025-11-17

**Authors:** Dan Li, Lei Wang, Hao Li, Xuhua Guan

**Affiliations:** 1 Institute of Infectious Disease Prevention and Control, Hubei Provincial Center for Disease Control and Prevention, Wuhan, China; 2 School of Economics and Management, China University of Geosciences, Wuhan, China; 3 Institute of Immunization Program, Daye Center for Disease Control and Prevention, Daye, China

**Keywords:** pertussis-containing vaccines, immunization services, accessibility, supply and demand perspective, influencing factors

## Abstract

**Background:**

China consistently ranked among the top three contributors to global pertussis cases from 2021 to 2024. However, limited research on the accessibility of pertussis vaccines from a supply–demand perspective has hindered effective control measures.

**Methods:**

A birth cohort-stratified survey was conducted in Hubei Province, China, from March to August 2024. Data on the geographical, awareness-related, and economic accessibility of pertussis vaccination services were collected using electronic questionnaires and field surveys, targeting vaccination staff (supply-side) and caregivers of eligible children (demand-side). Influencing factors were analyzed using the χ^2^ test and univariate and multivariate logistic regression analyses.

**Results:**

A total of 1,350 vaccination staff members from 914 clinics and 1,098 caregivers of eligible children across 17 prefecture-level cities were surveyed. Geographically, 80% of vaccination staff members reported that their clinic’s nearest clients lived within 2 km, and 89.89% of caregivers of eligible children traveled <30 min for vaccination. Average awareness rates were 72.92% for vaccination staff and 69.90% for caregivers of eligible children (*χ^2^* = 14.887, *p* < 0.05). Among vaccination staff, female individuals and those aged ≤30 years with college education, senior titles, or more than 15 years of experience showed ≥2-fold higher odds of having good awareness. Caregivers of eligible children who held a college degree, worked in medical professions, resided in urban residence, fulfilled maternal/grandmaternal roles, had an income of ≥100,000 CNY, or had infants aged ≤12 months showed 1.5–2.6-fold higher awareness. Economically, 93.11% of vaccination staff worked in clinics that provided free National Immunization Program (NIP) pertussis vaccines (DTaP), and 87.80% of caregivers of eligible children were willing to pay for non-NIP pertussis vaccines.

**Conclusion:**

The geographical and economic accessibility of pertussis vaccination services is relatively reasonable in Hubei Province, China. However, knowledge gaps still exist on both the supply and demand sides. Targeted training for older, male, or less-experienced vaccination staff, along with customized education for rural, low-income, male caregivers of eligible children, could help bridge these gaps and reduce the disease burden in China.

## 1 Introduction

Pertussis is a highly contagious acute respiratory infection caused by *Bordetella pertussis* and ranks among the 10 leading causes of disease burden in children under 10 years of age worldwide (1). High-income countries with historically high vaccine coverage—such as the United States, the United Kingdom, and Australia—have witnessed a “pertussis resurgence” with notable increases in cases (2). In recent years, China has reported an unprecedented level of pertussis cases: the National Notifiable Disease Reporting System showed an average annual increase of 14.26% in incidence between 2018 and 2022. A total of 38,295 cases were reported in 2022 (incidence rate of 2.71 per 100,000), representing the highest number of cases globally and the sixth highest incidence rate. The number of cases rose from 41,124 in 2023 to 476,690 (the highest number of cases globally) in 2024, with a 10.59-fold increase in cases over 2023 (3–5).

Vaccination with pertussis-containing vaccines is one of the most effective methods to prevent pertussis. In China, the currently licensed vaccines include DTaP (diphtheria, tetanus, and acellular pertussis combined vaccine), DTaP-IPV-Hib (pentavalent), and DTaP-Hib (quadrivalent). DTwP (whole-cell) was introduced into the National Immunization Program (NIP) in 1978; DTaP gradually replaced DTwP starting in 2007, and the transition was completed in 2013 (6). Pentavalent and quadrivalent vaccines have been available as non-NIP options since 2011 and 2013, respectively. With the State Council approval, the NIP DTaP schedule was modified on 1 January 2025: the first dose was moved from 3 months to 2 months of age, followed by subsequent doses at 4, 6, and 18 months, with a booster shot at 6 years of age (7). If children of appropriate age received a pertussis-containing non-NIP vaccine, they can be considered to have received the corresponding dose of the DTaP vaccine.

Successful immunization depends not only on vaccine safety and efficacy but also on the accessibility of immunization service (3). Previous studies have primarily focused on the survey of pertussis vaccine immunization services from either the supply side or the demand side, considering only a single dimension (8, 9). “Supply–demand coupling in vaccination services” is defined as the integrated investigation of vaccination service accessibility by simultaneously considering the perspectives of two key stakeholders: the supply side (vaccination staff who deliver vaccine services) and the demand side (caregivers of eligible children who seek and utilize these services). Notably, there is a lack of quantitative research that systematically evaluates pertussis vaccine service accessibility from a supply–demand coupling perspective, limiting the ability to identify systemic bottlenecks in vaccine service. Hubei Province, located in central China with 58.3 million residents across 185,900 km^2^, reported 24,014 pertussis cases (an 11.5-fold increase over 2023), ranking eighth nationally. To gain better insights into strategies for improving pertussis vaccine service delivery, we adopted a supply–demand coupling framework to evaluate three critical dimensions—geographical, awareness-related, and economic accessibility—of immunization services in Hubei Province, China.

## 2 Materials and methods

### 2.1 Study participants

Supply side: Vaccination staff members in Hubei Province. Eligible participants were healthcare staff involved in the vaccination process, including vaccinators, appointment/registration clerks, administrators, and physical-examination personnel, who had been working in vaccination services for at least 6 months.

Demand side: Primary caregivers (parents, grandparents, or other relatives) of children eligible for pertussis-containing vaccines in Hubei Province.

### 2.2 Survey design

A stratified sampling strategy was used, with sample sizes allocated in proportion to each prefecture’s 2023 birth cohort. Using the cross-sectional sample-size formula *n = Z^2^·p·(1-p)/d^2^* (*Z* = 1.96 for 95% CI, *d* = 0.03 margin of error, *p* = 0.5 assumed awareness), the minimum required sample was 1,007 vaccination staff members and 660 caregivers. Face-to-face electronic questionnaires were administered from March to August 2024. Each prefecture distributed the questionnaires throughout the region, and respondents voluntarily participated in the face-to-face survey until the required sample size was reached in all prefectures. Respondents participated randomly and voluntarily, with no mandated intervention. All interviewers received standardized training and used uniform scripts, while data completeness was verified in real time.

### 2.3 Survey instruments and key variables

Two structured questionnaires—“Questionnaire on Pertussis-Containing Vaccines for Vaccination Staff” and “Questionnaire on Pertussis-Containing Vaccines for Caregivers”—were developed based on the WHO Knowledge, Attitudes, and Practices (KAP) survey guide (10) and relevant domestic literature (11). Both questionnaires covered basic demographics, geographical accessibility, knowledge of pertussis-containing vaccines, attitudes, willingness to vaccinate, and economic factors. The questionnaires were pre-tested in March 2024 among 58 vaccination staff members and 23 caregivers in Huangshi and Xianning cities. Cronbach’s *α* was 0.80 for the vaccination staff version and 0.78 for the caregiver version, which meets the acceptable internal consistency standard (Cronbach’s α ≥ 0.7).

Geographical accessibility was evaluated using two metrics on the supply side: the nearest service distance and the longest service distance (service radius). On the demand side, geographical accessibility was assessed based on three dimensions: the distance from the caregiver’s home to the nearest vaccination clinic within the jurisdiction, the primary transportation mode, and the travel time required to reach the clinic.

The awareness rate of pertussis-containing vaccines for individual respondents was calculated as follows: (Number of correctly answered questions per respondent) / (Number of questions answered by the respondent). The overall average awareness rate was calculated as follows: [∑ (Number of correctly answered questions per respondent) /Total number of questions answered across all respondents] × 100%.

Additionally, the awareness level regarding knowledge of pertussis-containing vaccine was converted into a binary variable, categorized as “good awareness” (number of correctly answered questions ≥ 60% of the total questions) and “poor awareness” (number of correctly answered questions < 60% of the total questions).

Chi-squared tests were performed to analyze factors influencing the awareness rate. Univariate logistic regression and multivariate logistic regression analyses were then employed to identify factors affecting the level of awareness.

### 2.4 Statistical analysis

Data were analyzed using SPSS 22.0. Between-group comparisons of categorical variables (e.g., the awareness rate and the timeliness ratio) were conducted using the χ^2^ test, with a significance level (*α*) of 0.05.

## 3 Results

### 3.1 Supply-side accessibility

#### 3.1.1 Characteristics of the surveyed vaccination staff

A total of 1,350 vaccination staff members from 914 clinics were validly surveyed. The male-to-female ratio of the surveyed vaccination staff was 1:8, and 71.26% were aged 31–50 years. The majority of them (91.93%) were employed in hospitals (township health centers, community hospitals, or maternal and child health hospitals). The largest occupational group was the vaccinator (59.11%), followed by appointment/registration staff (17.04%). The majority held a junior college degree (44.15%) or a bachelor’s degree (41.33%), and 59.26% (800 individuals) had a nursing background. Junior- or intermediate-level professional titles accounted for 93.70% of the sample. Work experience of ≤5 years was the most common (37.63%). Among the respondents, 96% worked in clinics that provided pertussis-containing vaccines, including the NIP DTaP vaccine (93.11%), non-NIP quadrivalent vaccine (66.22%), and non-NIP pentavalent vaccine (59.19%). See Supplementary Table 1.

#### 3.1.2 Supply-side geographical accessibility

Among the 1,350 vaccination staff members investigated, 30% reported that their clinic’s nearest clients were within 1 km, 50.37% were 1–2 km away, and 19.63% were more than 2 km away. In terms of service radius: nearly 6 in 10 clinics (59.33%) extend beyond 5 km, almost a quarter (23.33%) span 2–5 km, 10.07% are within 1 km, and 7.26% are between 1 and 2 km.

#### 3.1.3 Supply-side knowledge and influencing factors

Among the 1,350 vaccination staff members, the average awareness rate regarding pertussis-containing vaccines was 72.92%. The item “Which of the following vaccines can prevent pertussis?” achieved the highest accuracy (92.67%), whereas the item “What is the earliest age (in months) at which pertussis prevention can begin under the current NIP and non-NIP schedules” yielded the lowest accuracy (53.11%), see Table 1.

**Table 1:** Awareness of pertussis-containing vaccines among vaccination staff in Hubei Province, China, 2024.

Items	No. of awareness	Awareness rate (%)
Is pertussis a statutorily notifiable infectious disease in China?	1,188	88.00
What has been the trend of pertussis incidence in China over the past year?	1,048	77.63
Which population groups are susceptible to pertussis?	718	53.19
Which of the following vaccines can prevent pertussis?	1,251	92.67
What is the earliest age (in months) at which pertussis prevention can begin under the current NIP and non-NIP schedules?	717	53.11
Average	984	72.92

Factors influencing the awareness rate showed that female individuals demonstrated higher awareness than male individuals (73.66% vs. 67.02%; *χ^2^* = 14.978, *p* < 0.05). Age-specific rates peaked at 75.09% among vaccination staff aged 31–40 years, followed by 72.79% in those aged 41–50 years (*χ^2^* = 17.268, *p* < 0.05). Educational attainment exhibited a strong gradient: 87.20% of individuals holding master’s degrees or above, 76.49% among those with bachelor’s degrees, 71.88% among those with associate degrees, and 62.81% among those with a high school education or below (*χ^2^* = 76.804, *p* < 0.05). Professional rank displayed a similar trend: 85.71% at the senior level, 80.00% at the associate senior level, 75.46% at the intermediate level, and 69.93% at the junior level (*χ^2^* = 37.859, *p* < 0.05). Years of experience in immunization work were not associated with awareness (*χ^2^* = 4.551, *p* > 0.05), see Table 2.

**Table 2:** Factors affecting the awareness rate regarding pertussis-containing vaccines among vaccination staff in Hubei Province, China, 2024.

Characteristic	No.	%	No. of questions	No. of awareness	Awareness rate (%)	*χ^2^*	*p*
Sex							
Male individuals	151	11.19	755	506	67.02	14.978	0.000
Female individuals	1,199	88.81	5,995	4,416	73.66		
Age (year)			0				
>50	189	14.00	945	643	68.04	17.268	0.000
31–40	485	35.93	2,425	1821	75.09		
41–50	477	35.33	2,385	1736	72.79		
≤30	199	14.74	995	722	72.56		
Education			0				
Senior high school or below	171	12.67	855	537	62.81	76.804	0.000
Junior college	596	44.15	2,980	2,142	71.88		
Bachelor’s degree	558	41.33	2,790	2,134	76.49		
Master’s degree or above	25	1.85	125	109	87.20		
Professional title							
Junior	697	51.63	3,485	2,437	69.93	37.859	0.000
Intermediate	568	42.07	2,840	2,143	75.46		
Associate senior	78	5.78	390	312	80.00		
Senior	7	0.52	35	30	85.71		
Years in immunization practice							
0–5	508	37.63	2,540	1841	72.48	4.551	0.336
>5–10	350	25.93	1750	1,266	72.34		
>10–15	236	17.48	1,180	880	74.58		
>15–20	88	6.52	440	333	75.68		
>20	168	12.44	840	602	71.67		
Total	1,350	100.00	6,750	4,922	72.92		

After conducting a univariable logistic regression analysis, the subsequent multivariate logistic regression analysis showed that female immunization staff had 2.12-fold higher odds of good awareness than male staff (95% CI 1.33–3.39); awareness declined significantly after age 40 (OR 0.54 for 41–50 years and 0.29 for >50 years vs. ≤30 years); college and bachelor’s degree holders were 2.11 and 2.48 times more likely to have good awareness than those with a senior high school education or less (95% CI 1.35–3.28 and 1.47–4.19, respectively); intermediate and associate-senior titles also conferred higher awareness, and staff with >15 years of experience were approximately twice as likely to have good awareness compared to those with ≤5 years, see Table 3.

**Table 3:** Multivariable logistic analysis of the awareness levels regarding pertussis-containing vaccines among vaccination staff in Hubei Province, China, 2024.

Variable	Classification	Partial regression coefficient β	Standard error	Wald χ^2^ value	*p-*value	OR	95%CI	
							Lower	Upper
Sex	Male individuals	Ref						
	Female individuals	0.75	0.24	9.93	0.00	2.12	1.33	3.39
Age (year)	≤30	Ref						
	31–40	−0.07	0.28	0.06	0.81	0.93	0.54	1.62
	41–50	−0.62	0.31	4.05	0.04	0.54	0.30	0.98
	>50	−1.23	0.37	10.74	0.00	0.29	0.14	0.61
Education	Senior high school or below	Ref						
	Junior college	0.74	0.23	10.91	0.00	2.11	1.35	3.28
	Bachelor’s degree	0.91	0.27	11.66	0.00	2.48	1.47	4.19
	Master’s degree or above	14.38	575.38	0.00	0.98	1763090.31	0.00	.c
Professional title	Junior	Ref						
	Intermediate	0.57	0.19	9.07	0.00	1.77	1.22	2.56
	Associate senior	1.20	0.47	6.56	0.01	3.34	1.33	8.39
	Senior	14.74	0.00			2514516.96	2514516.96	2514516.96
Years in immunization practice	0–5	Ref						
	>5–10	0.09	0.21	0.19	0.66	1.10	0.73	1.66
	>10–15	0.44	0.26	2.88	0.09	1.56	0.93	2.59
	>15–20	0.97	0.41	5.59	0.02	2.63	1.18	5.87
	>20	0.79	0.31	6.51	0.01	2.21	1.20	4.06

#### 3.1.4 Supply-side attitude

A total of 95.11% of vaccination staff endorsed the statement that early pertussis vaccination can prevent infection in young infants and avert progression to severe disease or death; 86.52% prioritized communicating the elevated risk of pertussis and its severe course in infants under 6 months of age when counseling caregivers; 84.37% preferred verbal explanation as the primary method to clarify the recommended pertussis-containing vaccine schedule; 72.07% indicated that they consistently disseminate evidence on the benefits of early pertussis vaccination during academic exchanges or conferences; and 73.11% recommended adding a preschool booster dose to the existing pertussis immunization program.

#### 3.1.5 Supply-side behavior

A total of 70.3% of vaccination staff worked in clinics that held sessions, including 26.0% worked in clinics that held sessions once every 6–12 months, 17.5% worked in clinics that held sessions every 3–6 months, 20.4 % worked in clinics that held sessions every 1–3 months, and 6.4 % worked in clinics that held sessions every 0.5–1 month. For reminder modalities (multiple selections permitted), telephone calls were used by 97.0% of the respondents, 75.6% used parent-group chat notifications, 74.1% used Short Message Service, 60.7% used WeChat official account messages, 42.4% made announcements in parent classes, and 5.9% used other means. Regarding proactive outreach after a missed vaccination, 52.3% of the respondents contacted parents 1 week–1 month after the due date, 21.6% reached out at 1–3 months, 21.0% made contact within 1 week, 3.9% followed up after 3 months, and 1.2% reported no active follow-up.

### 3.2 Demand-side accessibility

#### 3.2.1 Characteristics of the surveyed caregivers of age-eligible children

Among the 1,098 caregivers of age-eligible children, the male-to-female ratio was 1:3, and 64.02% were aged 31–50 years. Mothers constituted 69.13% of the sample. Educational attainment was predominantly bachelor^’^s degree (35.52%) and junior college (33.33%). A majority (53.92%) worked as medical professionals. Annual household income was below 100,000 CNY for 73.50% of families. The majority of children were ≥12 months old (64.12%), resided in urban areas (57.29%), and were the first-born in their families (59.02%). See Supplementary Table 2.

#### 3.2.2 Demand-side geographical accessibility

Among the 1,098 caregivers of age-eligible children, 57.29% lived within 3 km of the nearest vaccination clinic, 45.63% could reach it in <10 min, and 44.26% required 10–30 min. A total of 38.07% usually traveled by private car, while 30.24% walked. Overall, 61.02% completed registration within 10 min, and 53.37% had their child vaccinated within 10 min after registration.

#### 3.2.3 Demand-side knowledge and influencing factors

The average awareness rate regarding pertussis-containing vaccines was 69.90% (4,605/6,588) among the surveyed caregivers of age-eligible children, which was lower than that of vaccination staff (72.92%; *χ^2^* = 14.887, *p* < 0.05). The highest accuracy pertained to the statement “DTaP is a National Immunization Program vaccine” (85.15%), whereas the lowest accuracy was observed for the question “At what is the earliest age, in months, can pertussis prevention begin with currently available vaccines?” (40.35%; Table 4).

**Table 4:** Awareness of pertussis-containing vaccines among caregivers of children in Hubei Province, China, 2024.

Items	No. of people aware	Awareness rate (%)
Is pertussis a notifiable infectious disease in China?	876	79.78
Which vaccines can prevent pertussis?	804	73.22
What is the earliest age at which pertussis prevention can begin with currently available vaccines?	443	40.35
Awareness of NIP versus non-NIP classification		
DTaP	935	85.15
DTaP/Hib	766	69.76
DTaP-IPV/Hib	781	71.13
Average	768	69.90

Factors affecting analysis showed that the awareness rate was higher in female individuals (71.09% vs. 66.09% in male individuals), healthcare workers (77.17% vs. 61.40%), degree holders (72.59–78.13%) compared to those with a senior high school education or below (56.75%), urban residents (76.26%) versus peri-urban (70.94%) or rural residents (58.33%), grandparents (75.00%) versus parents (65.41–71.09%); households earning ≥100,000 CNY (77.15% vs. 67.29%), caregivers of boys (70.96% vs. 68.59% for girls); and caregivers of children aged 13–24 months (74.64%) compared to those >72 months (66.87%). All *p*-values above were <0.05 (Table 5).

**Table 5:** Factors affecting the awareness rate regarding pertussis-containing vaccines among caregivers of eligible children in Hubei Province, China, 2024.

Characteristic	No.	%	No. of questions	No. of people aware	Awareness rate (%)	*χ^2^*	*p*
Parent’s sex							
Male individuals	262	23.86	1,572	1,039	66.09	14.212	0.000
Female individuals	836	76.14	5,016	3,566	71.09		
Parental age (year)							
≤30	286	26.05	1716	1,217	70.92	5.305	0.151
31–40	477	43.44	2,862	1960	68.48		
41–50	226	20.58	1,356	970	71.53		
>50	109	9.93	654	458	70.03		
Parental education							
Senior high school or below	326	29.69	1956	1,110	56.75	245.003	0.000
Junior college	366	33.33	2,196	1,594	72.59		
Bachelor’s degree	390	35.52	2,340	1826	78.03		
Master’s degree or above	16	1.46	96	75	78.13		
Parental occupation							
Medical professional	592	53.92	3,552	2,741	77.17	193.511	0.000
Other non-medical occupation	506	46.08	3,036	1864	61.40		
Residence							
Urban	629	57.29	3,774	2,878	76.26	208.698	0.000
Rural	356	32.42	2,136	1,246	58.33		
Urban–rural fringe	113	10.29	678	481	70.94		
Relationship to the child							
Mother	759	69.13	4,554	3,186	69.96	22.088	0.000
Father	185	16.85	1,110	726	65.41		
Grandparent	154	14.03	924	693	75.00		
Annual household income (CNY)							
<100,000	807	73.50	4,842	3,258	67.29	59.314	0
≥100,000	291	26.50	1746	1,347	77.15		
Child’s sex							
Male individuals	605	55.10	3,630	2,576	70.96	4.353	0.037
Female individuals	493	44.90	2,958	2029	68.59		
Child’s age group (months)							
≤12	429	39.07	2,574	1852	71.95	23.524	0.000
13–24	117	10.66	702	524	74.64		
25–72	305	27.78	1830	1,238	67.65		
>72	247	22.50	1,482	991	66.87		
Total	1,098	100.00	6,588	4,605	69.90		

After univariable logistic regression analysis, multivariate logistic regression analysis showed that caregivers with junior college, bachelor’s, or postgraduate education were 1.54, 2.14, and 2.04 times more likely to have good awareness of pertussis-containing vaccines than those with senior high school education or less (95% CI 1.07–2.23, 1.41–3.25, and 0.58–7.12, respectively). Medical professionals scored 2.14-fold higher than those in other occupations (95% CI: 1.57–2.93). Urban and urban-fringe residents outperformed the rural residents (OR 2.32 and 1.98; 95% CI 1.68–3.22 and 1.23–3.20). Mothers and grandmothers had 1.56- and 2.63-fold higher awareness than fathers (95% CI 1.09–2.24 and 1.25–5.55). Annual household income of CNY 100000–200,000 conferred 1.51-fold higher odds (95 %CI 1.02–2.23), and caregivers of infants ≤12 months were 1.71 times more aware than those of children >72 months (95% CI 1.16–2.50; Table 6).

**Table 6:** Multivariable logistic analysis of the awareness level regarding pertussis-containing vaccines among caregivers of eligible children in Hubei Province, China, 2024.

Variable	Classification	Partial regression coefficient β	Standard error	Wald χ^2^ value	*p-*value	OR	95%CI	
							Lower	Upper
Parental education	Senior high school or below	Ref						
	Junior college	0.43	0.19	5.30	0.02	1.54	1.07	2.23
	Bachelor’s degree	0.76	0.21	12.86	0.00	2.14	1.41	3.25
	Master’s degree or above	0.71	0.64	1.24	0.26	2.04	0.58	7.12
Parental occupation	Other non-medical occupation	Ref						
	Medical professional	0.76	0.16	22.74	0.00	2.14	1.57	2.93
Residence	Urban	0.84	0.17	25.85	0.00	2.32	1.68	3.22
	Rural	Ref						
	Urban–rural fringe	0.68	0.24	7.84	0.01	1.98	1.23	3.20
Relationship to the child	Mother	0.45	0.19	5.81	0.02	1.56	1.09	2.24
	Father	Ref						
	Grandfather	−0.02	0.39	0.00	0.96	0.98	0.45	2.13
	Grandmother	0.97	0.38	6.48	0.01	2.63	1.25	5.55
Annual household income (CNY)	<100,000	Ref						
	100,000–200,000	0.41	0.20	4.33	0.04	1.51	1.02	2.23
	200,000–500,000	−0.01	0.41	0.00	0.98	0.99	0.44	2.22
	≥500,000	0.72	0.70	1.08	0.30	2.06	0.53	8.05
Child’s age group (months)	≤12	0.53	0.19	7.51	0.01	1.71	1.16	2.50
	13–24	0.32	0.27	1.44	0.23	1.38	0.82	2.34
	25–72	−0.05	0.20	0.06	0.81	0.95	0.65	1.41
	>72	Ref						

#### 3.2.4 Demand-side behavior

Among the 1,098 surveyed caregivers, 93.08% had already initiated pertussis-containing vaccine series for their child, with 55.65% receiving DTaP, 11.84% the quadrivalent vaccine, and 25.59% the pentavalent vaccine. The remaining 6.92% had not yet administered the first dose; the most frequently cited reasons were lack of notification from the clinic (28.95%), the child not yet being due (25%), forgetting the appointment (17.11%), unwillingness to vaccinate (6.58%), and temporary vaccine unavailability (6.58%). Delayed first-dose administration was reported by 10.20% of caregivers, primarily attributed to the child’s illness (34.82%), forgetting the scheduled date (26.79%), delayed notification from the clinic (12.50%), temporary vaccine shortage (11.61%), and the perception that delay was inconsequential (5.36%). During clinic visits, 83, 78, and 77% of caregivers reported that vaccination staff introduced DTaP, quadrivalent, and pentavalent vaccines, respectively; for appointment scheduling, 58% used on-site booking, 22% online booking, and 20% again on-site booking.

A total of 64.11% of the surveyed caregivers had previously attended vaccine-education sessions held by their local clinic, 23.04% had not attended owing to lack of notification, and 12.84% had not attended for personal reasons. Caregivers who attended the vaccine-education session were significantly more knowledgeable about pertussis-containing vaccines than those who did not (Awareness rate: 72.54% vs. 65.19%, *χ^2^* = 38.94, *p* < 0.05) and were also more likely to have their child vaccinated on time (On-time vaccination rate: 94.32% vs. 81.73%, *χ^2^* = 43.73, *p* < 0.05).

#### 3.2.5 Demand-side economic accessibility

A total of 87.80% (964 caregivers) expressed their willingness to pay out of pocket for their children to receive non-NIP vaccines. Specifically, 43.17% (474 caregivers) were willing to spend no more than 500 yuan for one dose of a non-immunization program vaccine, and 44.63% (490 caregivers) preferred to consider cost-effectiveness and spend more than 500 yuan.

## 4 Discussion

Effective immunization programs remain the simplest and most powerful tools for controlling and preventing vaccine-preventable diseases (12, 13). Nevertheless, the WHO estimates that nearly 19 million children worldwide fail to receive all recommended doses annually, with the majority concentrated in low- and middle-income countries (14, 15). This study adopted a supply–demand coupling perspective to systematically evaluate the accessibility of pertussis vaccine immunization services in Hubei Province, China, covering three core dimensions: geographical, awareness-related, and economic accessibility. As one of the few studies to integrate both supply- and demand-side data for pertussis vaccination service assessment in central China, it provides empirical insights for optimizing pertussis control strategies amid the country’s ongoing pertussis resurgence. The findings reveal that while the overall accessibility framework is relatively sound, critical knowledge gaps persist on both sides, and targeted interventions are needed to address demographic and socioeconomic disparities.

Geographical accessibility is the main indicator to measure and evaluate the equity, efficiency, and quality of the health service system. On the supply side, 96% of clinics stocked pertussis-containing vaccines, 80% were within 2 km of the nearest residential area, and 59.33% maintained a service radius exceeding 5 km—indicating broad coverage that effectively reaches dispersed populations, including suburban and rural residents. However, on the demand side, 57% of caregivers lived within 3 km of the nearest vaccination clinic, and 46% could reach it in <10 min, considerably lower than a hepatitis B study among adults in Hebei Province, where 93% of adults lived less than 1 km from a vaccination site and 70% could reach it within 5–10 min (16). The above results show that although Hubei Province has a solid supply foundation for pertussis vaccination services, there is still significant room for improvement in terms of the convenience with which residents can actually access these services. In the future, efforts can be made to narrow gaps in cases of high-quality vaccine service accessibility by further optimizing the spatial layout of vaccination sites and increasing the number of primary-level vaccination sites.

The awareness rate regarding pertussis-containing vaccines was 72.9% among vaccination staff and 69.9% among caregivers of eligible children, both falling short of the WHO’s ≥ 80% confidence benchmark (15). This echoes qualitative work in Sichuan, Guangdong, and Henan, where “knowledge gaps, not supply shortages” were the dominant barrier (17). The awareness rate was strongly influenced by sociodemographic factors. On the supply side, female individuals aged 31–40 years with graduate degrees or senior titles scored 15–18 percentage points higher in vaccine awareness than their peers—a gradient comparable to the provider-literacy findings in the GBD 2023 multi-country analysis (18). On the demand side, the steep urban–rural (76% vs. 58%) and income-based (≥100,000 CNY vs. <100,000 CNY: 77% vs. 67%) awareness rate gaps were also reported in sub-Saharan Africa (19) and in low-uptake provinces in southern China (20). Novel for this region, grandparents achieved the highest awareness rate (75%), surpassing fathers (65%) and mothers (71%), aligning with observations of grandparent gatekeeping in Japan (21). Caregivers of boys also scored significantly higher than those of girls, extending the sex-specific knowledge gap reported in HPV studies (22). It is noted that more than half of the respondents were medical professionals, who exhibited higher awareness compared to non-medical counterparts (77% vs. 61%) and could inflate the awareness level due to their occupational exposure and health literacy.

When the awareness level of pertussis-containing vaccine knowledge was converted into a binary variable (“good awareness” and “poor awareness”), our results were as follows: among vaccination staff, good awareness of pertussis vaccines was at least twice as common in female individuals, those aged ≤30 years, degree holders, senior staff, and workers with >15 years of experience; among caregivers, awareness increased 1.5–2.6 fold if they had a degree, worked in healthcare, lived in cities, were the child’s mother/grandmother, earned ≥100,000 CNY/year, or cared for an infant ≤12 months. Compared to vaccination staff with lower education or less experience, those with higher education and longer tenure tend to access more comprehensive training and updated information—consistent with a study that linked providers’ knowledge and experience to better vaccine-related recommendations (23). Similarly, caregivers who had higher education or worked in medical professions gain more daily exposure to health knowledge, which explains their higher awareness, as supported by Brazilian research highlighting the impact of occupation-related knowledge on vaccine hesitancy (24). To narrow awareness gaps, targeted measures are needed: design interactive, technology-driven training for younger vaccination staff, organize community-based simplified education for rural/low-income caregivers, and use social media to expand educational outreach to underserved groups.

Regarding health education, caregivers who attended vaccine-education sessions showed significantly higher awareness of pertussis-containing vaccines (72.54% vs. 65.19%, χ^2^ = 38.94, *p* < 0.05) and a notably higher rate of on-time vaccination for their children (94.32% vs. 81.73%, χ^2^ = 43.73, *p* < 0.05). This result highlights the key role of vaccine-education sessions in improving caregivers’ knowledge and promoting standardized vaccination. Previous research has also emphasized the significance of such educational initiatives. A study in Pakistan demonstrated that a simple educational intervention designed for low-literate populations improved DPT-3/hepatitis B vaccine completion rates by 39% (25). Our study also showed that 70% of vaccination staff worked in clinics that conducted parent-education sessions, with only 6.4% holding sessions every 0.5–1 month. It is recommended that local health departments increase the frequency of parent-education sessions, aiming for at least monthly sessions in the vaccination clinics.

Although 87.8% of caregivers declared willingness to pay, 43% set a ceiling of ≤500 CNY—just below the current private-market price (~600 CNY). This mirrors the price-elasticity breakpoint identified in Shanghai during the COVID-19 pandemic (26) and Kenya’s M-SIMU trial, where conditional cash transfers raised non-NIP coverage by 11% (27). These findings indicate that relevant authorities should integrate fiscal and healthcare-insurance resources, strengthen reimbursement for preventive medications to boost caregivers’ willingness to vaccinate, and negotiate prices of costly vaccines through competitive bidding so they can be included in centralized procurement catalogs. For example, Hubei Province provides free domestic bivalent HPV vaccination to girls aged 13-14 in 2024 and 2025. This initiative is funded primarily by local government budgets, supported by extensive public-education campaigns, and benefits from high public awareness and acceptance of the vaccine, thereby raising both awareness and uptake.

Unvaccinated status on the demand side was driven mainly by “lack of notification” (29%) and “forgetting” (25%), rather than vaccine refusal, consistent with the knowledge–behavior gap described in rural southwest China (28) and among Nigerian caregivers (19). Merely 52% actively followed up on delayed doses, a rate lower than the 75% achieved with SMS reminders in Taiwan (29). This underscores that targeted, low-literacy communication delivered via trusted doctors is more urgent than tackling traditional hesitancy (30). The finding indicates that vaccination behavior on the demand side is, to some extent, dependent on the supply side, reconfirming that digital nudges and outreach vaccination services on the supply side can improve vaccination behavior on the demand side.

This study had some limitations. First, the sample was limited to Hubei Province, and the results may not be applicable to other regions with greater economic disparities and cultural differences. Second, the proportion of medical practitioners among caregivers was relatively high, which may have inflated the awareness level. Third, the questionnaire survey may have limitations, such as potential issues with sampling representativeness, recall bias, and self-report bias; therefore, the results need to be integrated with the vaccination information management system to improve the scientific basis for policy.

## 5 Conclusion

Pertussis vaccination services in Hubei Province have relatively sound geographical and economic accessibility: most residents can easily reach clinics, free National Immunization Program vaccines are widely available, and most caregivers are willing to pay for non-program vaccines. However, knowledge gaps exist for both vaccination staff and caregivers of children. Vaccination staff members who are male, older, less educated, or less experienced have lower awareness; caregivers of eligible children who are rural residents, have lower income, are male, or care for older children also lack sufficient vaccine knowledge. To narrow these gaps and reduce China’s pertussis burden, targeted measures are needed: provide customized training for vulnerable staff groups, expand rural community education with easy materials, and deliver accurate vaccine information to male caregivers and low-income families.

## Data Availability

The original contributions presented in the study are included in the article/Supplementary material, further inquiries can be directed to the corresponding author.
