# Supplementary material for: Accessibility of pertussis vaccine immunization services in Hubei Province from a supply–demand coupling perspective
Source: Front Public Health. 2025 Nov 17;13:1669984. doi: 10.3389/fpubh.2025.1669984 (PMC12667180; doi:10.3389/fpubh.2025.1669984)
Supplement: Supplementary file 1 [file Data_Sheet_1.docx]

Supplemental data

| Supplemental Table 1. Basic characteristics of survey vaccination staffs on pertussis-containing vaccines in Hubei Province, China, 2024. | | | |
| --- | --- | --- | --- |
| Characteristic | Category | No. of respondents | % |
| Sex | Female | 1199 | 88.81 |
|  | Male | 151 | 11.19 |
| Age (years) | ≤30 | 199 | 14.74 |
|  | 31-40 | 485 | 35.93 |
|  | 41-50 | 477 | 35.33 |
|  | ＞50 | 189 | 14.00 |
| Institutions | Hospital | 1241 | 91.93 |
|  | CDC | 109 | 8.07 |
| Job type | Vaccinator | 798 | 59.11 |
|  | Appointment/Registration staff | 230 | 17.04 |
|  | Administrator | 131 | 9.70 |
|  | Physical examination personnel | 67 | 4.96 |
|  | Other | 124 | 9.19 |
| Education | High school or below | 171 | 12.67 |
|  | Junior college | 596 | 44.15 |
|  | Bachelor | 558 | 41.33 |
|  | Master or above | 25 | 1.85 |
| Professional background | Nursing | 800 | 59.26 |
|  | Clinical medicine | 238 | 17.63 |
|  | Public health | 230 | 17.04 |
|  | Other medical | 55 | 4.07 |
|  | Non-medical | 27 | 2.00 |
| Professional title | Junior | 697 | 51.63 |
|  | Intermediate | 568 | 42.07 |
|  | Associate senior | 78 | 5.78 |
|  | Senior | 7 | 0.52 |
| Years in vaccination-related work | 0–5 | 508 | 37.63 |
|  | 6–10 | 350 | 25.93 |
|  | 10–15 | 236 | 17.48 |
|  | 15–20 | 88 | 6.52 |
|  | Over 20 | 168 | 12.44 |

| Supplemental Table 2. Characteristics of surveyed caregivers of age-eligible children on pertussis-containing vaccines in Hubei Province, China, 2024. | | | |
| --- | --- | --- | --- |
| Characteristic | Category | No. of respondents | % |
| Sex | Female | 836 | 76.14 |
|  | Male | 262 | 23.86 |
| Age (years) | ≤30 | 286 | 26.05 |
|  | 31-40 | 477 | 43.44 |
|  | 41-50 | 226 | 20.58 |
|  | ＞50 | 109 | 9.93 |
| Relationship to the child | Mother | 759 | 69.13 |
|  | Father | 185 | 16.85 |
|  | Grandmother | 59 | 5.37 |
|  | Grandfather | 40 | 3.64 |
|  | Other | 55 | 5.01 |
| Education | Senior high school or below | 326 | 29.69 |
|  | Junior college | 366 | 33.33 |
|  | Bachelor’s degree | 390 | 35.52 |
|  | Master’s degree or above | 16 | 1.46 |
| Occupation | Medical professional | 592 | 53.92 |
|  | Enterprise employee or industrial worker | 83 | 7.56 |
|  | Household duties or unemployed | 78 | 7.10 |
|  | Farmer or fisher | 52 | 4.74 |
|  | Civil servant or public-institution employee | 51 | 4.64 |
|  | Self-employed | 51 | 4.64 |
|  | Preschool or primary/secondary school staff | 9 | 0.82 |
|  | Infant/toddler care provider | 3 | 0.27 |
|  | Other | 179 | 16.30 |
| Annual household income (CNY) | < 100,000 | 807 | 73.50 |
|  | 100,000–199,999 | 242 | 22.04 |
|  | 200,000–499,999 | 34 | 3.10 |
|  | ≥ 500,000 | 15 | 1.37 |
| Child’s age in months | 0 to < 2 months | 63 | 5.74 |
|  | 2 to < 3 months | 43 | 3.92 |
|  | 3 to < 6 months | 165 | 15.03 |
|  | 6 to < 12 months | 123 | 11.20 |
|  | ≥ 12 months | 704 | 64.12 |
| Child’s sex | Male | 605 | 55.10 |
|  | Female | 493 | 44.90 |
| Residence | Urban | 629 | 57.29 |
|  | Rural | 356 | 32.42 |
|  | Urban–rural fringe | 113 | 10.29 |
| Birth order | 1 | 648 | 59.02 |
|  | 2 | 432 | 39.34 |
|  | 3 | 18 | 1.64 |
